# A Survey of 6DoF Object Pose Estimation Methods for Different Application Scenarios

**DOI:** 10.3390/s24041076

**Published:** 2024-02-07

**Authors:** Jian Guan, Yingming Hao, Qingxiao Wu, Sicong Li, Yingjian Fang

**Affiliations:** 1 Key Laboratory of Opto-Electronic Information Processing, Chinese Academy of Sciences, Shenyang 110016, China; 2 Shenyang Institute of Automation, Chinese Academy of Sciences, Shenyang 110016, China; 3 Institutes for Robotics and Intelligent Manufacturing, Chinese Academy of Sciences, Shenyang 110169, China; 4 University of Chinese Academy of Sciences, Beijing 100049, China

**Keywords:** object pose estimation, deep learning, 6DoF pose, computer vision

## Abstract

Recently, 6DoF object pose estimation has become increasingly important for a broad range of applications in the fields of virtual reality, augmented reality, autonomous driving, and robotic operations. This task involves extracting the target area from the input data and subsequently determining the position and orientation of the objects. In recent years, many new advances have been made in pose estimation. However, existing reviews have the problem of only summarizing category-level or instance-level methods, and not comprehensively summarizing deep learning methods. This paper will provide a comprehensive review of the latest progress in 6D pose estimation to help researchers better understanding this area. In this study, the current methods about 6DoF object pose estimation are mainly categorized into two groups: instance-level and category-level groups, based on whether it is necessary to acquire the CAD model of the object. Recent advancements about learning-based 6DoF pose estimation methods are comprehensively reviewed. The study systematically explores the innovations and applicable scenarios of various methods. It provides an overview of widely used datasets, task metrics, and diverse application scenarios. Furthermore, state-of-the-art methods are compared across publicly accessible datasets, taking into account differences in input data types. Finally, we summarize the challenges of current tasks, methods for different applications, and future development directions.

## 1. Introduction

Object pose estimation is a key task in the field of computer vision, whose main goal is to accurately obtain a 6DoF (6 degrees of freedom) representation of the object pose in real-life scenes. This representation plays a key role in providing comprehensive information beyond two-dimensional understanding. Specifically, it encompasses three-degree-of-freedom rotation and three-degree-of-freedom translation. The significance of this task is that it can provide the precise spatial position of objects, so 6DoF object pose estimation is increasingly important for various applications of computer vision, such as virtual reality, augmented reality [1,2], automated driving [3], and robotic operation [4]. The continuous advancement of computer vision theory and the rapid development of related fields have prompted extensive and insightful research on 6DoF object pose estimation. Existing reviews make it difficult to summarize the latest research in this field, and this article will fill this gap by summarizing different approaches from recent years.

In robotics, the ability to perform complex tasks such as precise manipulations in dynamic or unpredictable environments is crucial. Many tasks require scene understanding or object operations; 6DoF pose estimation provides comprehensive information regarding both position and orientation, enabling robots to execute tasks like recognition, localization, and grasping with heightened precision and accuracy [5]. To accomplish its grasping tasks effectively, the robot needs to rely on information such as the position, contact, and occlusion captured by the camera. As the role of robots becomes increasingly more important for improving productivity and securing safety, 6DoF pose estimation has become a key technology. Beyond the grasping operations of robots, 6DoF pose estimation also plays a vital role in reducing environmental uncertainty. It works in two ways, including reducing the probability of robot collision and providing support for robot transportation or other activities [6,7].

Augmented reality (AR) and virtual reality (VR) technologies play an important role in the military, aerospace, education, entertainment and gaming, as well as many other fields. AR/VR systems can accurately track targets based on their poses, which is the foundation of immersive effects. One crucial aspect of this technique is 6DoF pose estimation, which enables the construction of a spatial mapping of the environment and provides information for the proper integration of AR/VR content. By estimating the precise 6DoF pose of the target, virtual objects can be rendered from different viewpoints, resulting in realistic visual effects in AR/VR experiences. In addition, precise alignment and interaction between virtual objects and the real world can be realized [8].

6DoF pose estimation plays an important role in many parts of autonomous driving tasks, including environment perception, obstacle detection, traffic condition prediction, and decision planning. It provides valuable information for trajectory planning and obstacle avoidance. With the rapid development of autonomous driving technology, the requirements for pose estimation precision are becoming increasingly high [9,10,11].

**Figure 1:**
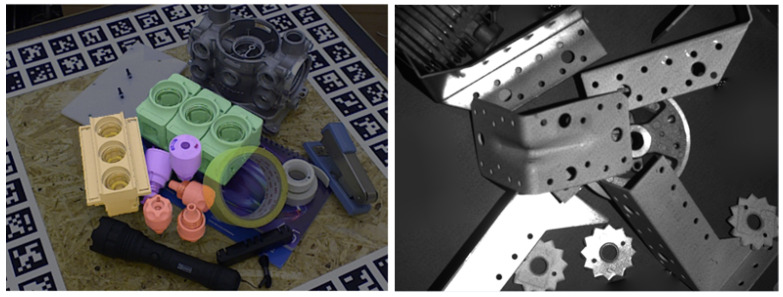
Different shapes (**left**) [12] and large occlusions (**right**) images [13].

Ideally, the pose estimation method should be able to handle objects with different shapes and textures and show robustness to large occlusions, noise, and changes in light. Figure 1 shows two possible situations. Furthermore, it should balance accuracy and efficiency, especially in real-time navigation tasks.

In the field of computer vision, the traditional approach involves extracting target features such as points, edges, and lines directly from input images or point clouds. These features are then matched with a reference image or model to perform pose estimation. The positional relationship can be solved by measuring the coordinates of multiple points under two spatial coordinate systems and utilizing the positional relationship between marker points as constraints. However, traditional methods rely on manually designed feature extraction, and their performance may be affected by noise, data quality, or other factors [14].

Over the past few years, advances in technology have made data collection easier, and deep learning methods have performed well in many areas of computer vision. With the rapid development of deep learning technology, 6DoF pose estimation based on deep learning [15,16,17] has significantly improved in terms of accuracy, robustness, and adaptability to different scenes. In various daily life scenarios, as well as industrial robotics tasks, the estimation of 6DoF object poses is of great significance and can bring great convenience to production and daily life.

**Figure 2:**
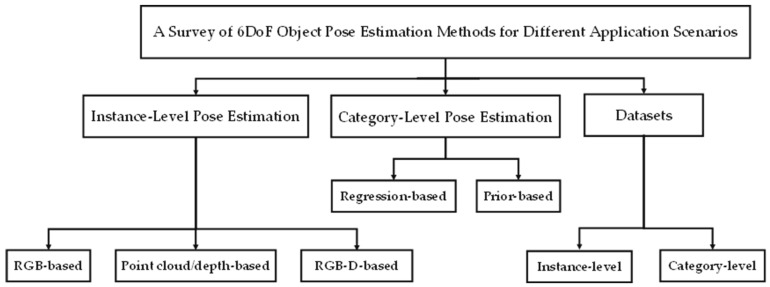
General Structure of the Survey.

Figure 2 illustrates the sections of this paper where the existing methods can be coarsely categorized into two main groups based on whether the object model is necessary for the training stage: instance-level pose estimation and category-level pose estimation.

Several reviews of this task already exist [4,6,14,18], but unlike these methods, here, we focus on methods based on deep learning, organize the paper according to different input data types or method types, and summarize commonly used public datasets in the field. We summarize the application scenarios of different methods, organize commonly used datasets in the field, and provide a comprehensive summary of methodologies. Additionally, the paper discusses future expectations based on different application scenarios, which can provide a valuable reference for subsequent research work.

## 2. Instance-Level 6DoF Object Pose Estimation

There has been significant research conducted on instance-level 6DoF object pose estimation. Depending on the input data, we classified these methods into three categories: RGB-based methods, point cloud or depth-based methods, and RGB-D-based methods. Then, we summarized the refinement methods.

### 2.1. RGB-Based Methods

**Figure 3:**
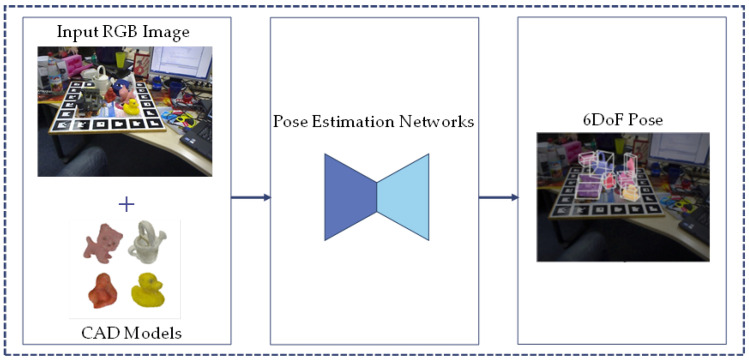
Schematic of a typical method for RGB-based pose estimation.

With the advancement of deep learning, RGB-based 6DoF pose estimation methods have made significant progress in both theoretical and practical aspects. Figure 3 shows an overview of the typical RGB-based pose estimation methods; RGB images provide rich visual information and scene texture features that enable deep learning networks to extract effective representations of object poses. Furthermore, the widespread use and affordability of RGB cameras contribute to their cost advantage.

The task of pose estimation from RGB images faces several challenges, such as occlusion and lack of texture information. In this section, following the common classification, RGB-based methods can be divided into three classes: regression-based methods, template-based methods, and feature-based methods.

#### 2.1.1. Regression-Based Methods

**Figure 4:**
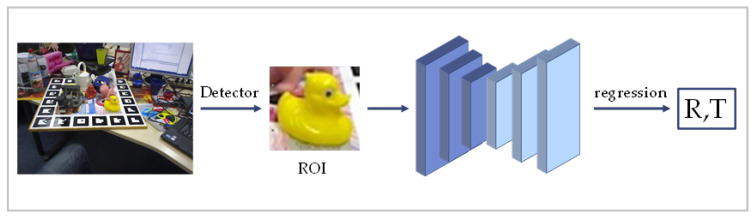
A typical flow of regression-based pose estimation methods.

One of the straightforward approaches is that the 6DoF object pose estimation is considered as a regression task, which directly predicts the poses from the input RGB images without intermediate keypoint representations. A typical simplified flow of the regression-based approach is shown in Figure 4.

PoseNet [19] is one of the first works to propose an end-to-end approach that introduces an end-to-end 6DoF pose estimation model using convolutional neural networks(CNNs). The model demonstrates the feasibility of deep learning for pose estimation by directly regressing the orientation and position of RGB images. However, this method is only used for human pose estimation.

PoseCNN [20] pioneers a direct learning approach for regression object 6D pose representation. The method treats rotations and translations separately, incorporates 2D centroid prediction based on hough voting, integrates additional prior knowledge for translation processing, and proposes a symmetric loss function for shape matching. It is worth mentioning that PoseCNN introduces the highly influential YCB-V dataset. However, the ICP algorithm [21] needs to be optimized for better accuracy, and the networks are specific and may have poor generalization. Overall, this is a classical work.

Deep-6DPose [22] extends the instance segmentation network Mask R-CNN [23] to pose estimation. It directly introduces a pose prediction branch into the framework, thus realizing an end-to-end regression-based pose estimation method. Different from PoseCNN [20], Deep-6Dpose does not require subsequent refinement steps, thus simplifying the process and improving efficiency. However, its performance decreases significantly when the pose changes significantly, and its accuracy still needs to be improved.

Hu et al. [24] proposes a segmentation-driven simple pose estimation network, which enables handling multiple objects occluding each other, even in the absence of texture. This method avoids the need for post-processing, but the estimation of small objects needs to be improved. Additionally, there is room for improvement in the network architecture and fusion strategies.

Another noteworthy contribution is YOLO-6D [25], which leverages the YOLO family [26,27,28,29]. YOLO-6D [25] converts the pose estimation problem into a nine keypoints regression task and utilizes the real-time framework of YOLO-V2 [30]. This approach has had a significant impact on subsequent research. As the YOLO family continues to evolve, using new YOLO frameworks may produce better results, but application in complex environments may be limited. NeRF [31] introduces a method for generating rendered images without relying on mesh models. Inspired by this, iNeRF [32] predicts the pose from a single RGB image, specifically targeting scenarios where object mesh models are not available during training or testing. iNeRF can be extended to category-level pose estimation, but it is susceptible to lighting and occlusion, and its real-time performance needs to be improved.

DeepIM [33] introduces iterative improvements by regressing the pose difference between the rendered pose assumptions and the input image. Building on DeepIM, CosyPose [34] improves by incorporating rotational continuity representation, symmetry-aware display processing, and network architecture updates. These improvements make it possible to recover a consistent scene across multiple views, thus facilitating 6D attitude estimation for multiple classes of objects. CosyPose obtained the best results on multiple datasets in the 2020 Benchmark for 6D Object Pose Estimation (BOP) Challenge [35] and many subsequent studies built upon it.

To better solve the occlusion problem, ZebraPose [36] proposes a method that uses a dense representation of the object surface with discrete descriptors. The method uses an encoder–decoder architecture for feature extraction and direct regression of pose without post-processing procedures. However, the method has limited generalization to instances with significant appearance differences, although it has also been shown that the dense correspondence method is more effective at solving problems related to occlusion.

Hai et al. [37] address the limitations of existing self-supervised methods, which often require additional depth [38,39] or segmentation mask information [40]. They propose a self-supervised pose optimization framework that employs a synthetic dataset generated from the 3D mesh of the target object. The network is trained solely on this dataset to obtain the initial pose, followed by rendering multiple synthetic images from different viewpoints. To bridge the domain gap between synthetic and real data, a pseudo-label-based optimization strategy is employed for refinement. However, this approach heavily relies on synthetic data for training initial poses, and there is room for improving the generalization of current methods.

To address the problem of the increasing runtime when performing multi-object tasks, EfficientPose [41] proposes an efficient end-to-end pose estimation method that utilizes two additional sub-networks for predicting translations and rotations, thereby reducing computational cost and eliminating post-processing steps. The method also proposes a data enhancement technique involving random rotation and scaling of images to improve generalization to small datasets. However, as EfficientPose relies on overall detection, it may be less effective in heavily occluded scenes.

GDR-Net [15] proposes a simple and effective method for geometrically guided pose estimation. It dynamically scales up the detection results of other methods as the inputs and utilizes intermediate representations of dense correspondences. A modified version of this method utilizes a more robust backbone network and was successful in the BOP2022 challenge [42], demonstrating impressive accuracy and speed.

These regression-based methods have made significant progress in RGB-based pose estimation. However, there are still some challenges, such as dealing with occlusion, coping with illumination variations, improving real-time performance, and enhancing generalization to different scenes and object appearances.

#### 2.1.2. Template-Based Methods

**Figure 5:**
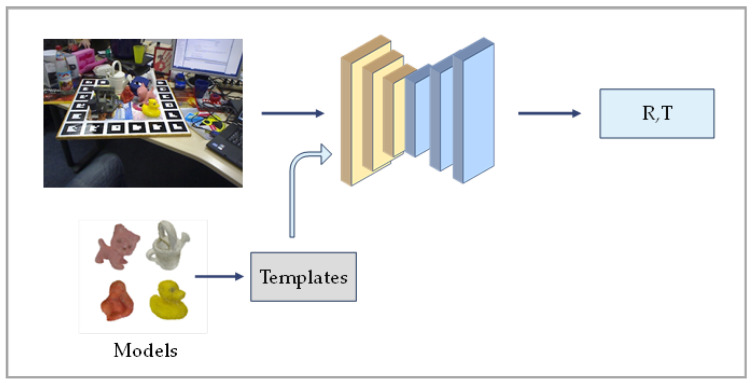
Typical flow of template-based pose estimation methods.

Template-based methods typically require finding the most similar template of the target, this search is conducted among many templates labeled with true poses, and then performing 6D pose estimation. Template matching is also a broad class of direct methods used for pose estimation. A typical simplified flow of the template-based approach is shown in Figure 5.

SSD-6D [43] is an approach that extends the 2D target detection network to handle the pose estimation task. It introduces a pose estimation branch during the detection process and decomposes the pose space of the model, using the multiple detection results obtained, performing a series of pose estimations as templates, and selecting an optimal hypothesis. Additionally, it treats rotational regression as a classification task, which improves the training and learning of symmetric objects. However, an inherent limitation of the method is its reliance on regressing the 2D bounding box corners. This reliance can result in decreased accuracy, particularly for heavily occluded objects. Furthermore, methods such as data augmentation need to be used to minimize the differences between synthetic images and real data.

LatentFusion [44] introduces a novel framework for the 6D pose estimation of unseen targets by leveraging learned 3D representations. The network is capable of rendering the target from any viewpoint and directly optimizing the pose of the input image. The method achieves this by training the network on a large dataset of 3D shapes, enabling it to reconstruct and render objects accurately. Moreover, the use of multiple views during modeling allows for robust observations, and the consistency across these views enables the construction of a canonical representation, resulting in improved generalization to unseen targets. However, it’s worth noting that LatentFusion’s iterative optimization process during inference can be computationally expensive. Additionally, the method is sensitive to occlusions in the input data, which can lead to significant performance degradation when occlusions are present.

DPOD [45] is a pose estimation method that combines detection and matching using a dense matching-based approach. In the first stage, a detector predicts 2D frames. The second stage refers to the voting-based PVNet [46] method for template matching. DPOD can estimate poses from a single RGB image without requiring perfect segmentation. It demonstrates robustness in handling occlusion and lighting changes. However, it may be less effective for objects that lack distinctive color features.

PoseRBPF [47] utilizes a Rao-Blackwellized particle filter that samples object poses and estimates the discretized distribution of each particle’s rotations using a pre-computed codebook. This method is effective at tracking object poses and is less susceptible to motion blur and occlusion. However, it may encounter difficulties when objects are heavily occluded or measurements deviate significantly from the synthetic training data. Furthermore, there is room for improvement in the discretized rotation representation in PoseRBPF.

OSOP [48] utilizes semantic segmentation to predict the mask of the visible parts, and renders 2D templates from various viewpoints, using the templates to first locate the approximate viewpoints, and then obtain the final pose after dense matching, which can be generalized to train unseen novel objects. It is applicable for tasks that need to be performed on new objects, although the domain gap between synthetic and real data needs to be handled.

Template-based methods have many advantages, including simplicity, speed, adaptability to changes in appearance, and better handling of weakly textured objects. It is a straightforward and intuitive method that can quickly detect and localize objects. However, template matching may face challenges in complex scenarios with occlusions, lighting variations, or objects lacking distinctive features. Incorporating local representations and addressing these challenges is crucial for robust template matching. Additionally, global matching in template matching can be influenced by the background and may perform poorly when matching real images of unseen objects [49,50]. Therefore, the use of local representations becomes necessary for template matching of unseen objects.

#### 2.1.3. Feature-Based Methods

**Figure 6:**
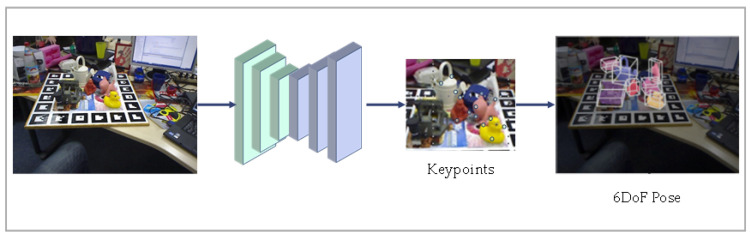
Typical flow of feature-based pose estimation methods.

The feature-based pose estimation method is widely used in the field of 6DoF pose estimation. The standard procedure involves extracting features from the input image, matching them with corresponding features in an existing 3D model, and then establishing the correspondence of 2D–3D coordinates using the Perspective-n-Point (PnP) algorithm. PnP is the corresponding method for solving 3D to 2D points. It describes how to estimate the pose of a camera when 3D space points and their positions are known. By leveraging the features extracted from the image and matching them with the features of the 3D model, this feature-based method establishes the 2D–3D relationship and enables accurate estimation of the target object’s 6D position. A typical simplified flow of the feature-based approach is shown in Figure 6.

The feature-based matching method has been extensively studied over time. Traditionally, feature points are extracted from two images, and then these feature points are compared to determine their correspondence. Global matching methods [51,52] perform well, with low computing power requirements, but they are sensitive to occlusion and noise, limiting their practical application. On the other hand, localized features are more robust when dealing with occlusion. Local feature extraction relies on feature detection and descriptors that should possess distinctiveness and invariance to certain transformations.

Traditional descriptors [53,54,55,56,57], such as SIFT [53], are manually designed and have certain limitations. They may not capture sufficient information, primarily describing geometric relationships, and can be less effective when the texture is not rich or the environment undergoes significant changes. In contrast, local feature detection and matching methods [58] based on deep learning have shown a better performance compared with traditional methods that rely on hand-crafted local features. These methods typically involve two steps: the first stage utilizes neural networks for feature extraction and obtaining 2D–3D correspondences, while the second stage solves the PnP problem. The differences between these methods mainly lie in how they establish the correspondence. These methods effectively leverage the advantages of CNN network structures, combining them with traditional computer vision techniques, and resulting in improved accuracy in pose estimation.

Pavlakos et al. [59] proposes a method that utilizes detected semantic keypoints to regress and compute the 6DoF pose in an end-to-end training fashion. This approach avoids the laborious process of point-by-point matching. However, it may be less effective when dealing with small objects and severe occlusion.

BB8 [60] utilizes segmentation methods to predict 3D boundary points based on 2D bounding boxes. It avoids the need for feature extraction and matching. Pose estimation is achieved by regressing the 2D coordinates of the inflection points of the projected 3D bounding box corners. BB8 demonstrates that the accurate and stable 3D pose estimation can be accomplished using only RGB information. The approach is extensible to new object categories without the need for predefined models. The estimation of symmetric objects has always been difficult. To solve this problem, BB8 explicitly handles it by range transformation and constraining object-labeled poses during training. This approach to handling symmetry has become more widely used in subsequent works [37,61]. However, it may be less effective for untextured objects.

To address the challenges posed by severe occlusion, PVNet [46] builds upon the symmetry handling approach introduced in [60]. The proposed pose estimation framework in PVNet [46] regresses pixel-level vectors that point to the keypoints. These vectors are then used for voting on the keypoints locations, resulting in a spatial probability distribution of the keypoints. Additionally, the network predicts pixel orientations, which allows it to focus more on the local features of the object and mitigate the effects of background clutter. PVNet [46] effectively solves the problem of occlusion and has laid the foundation for much of the subsequent work in the field.

EPOS [62] is a pose estimation method that takes into account the symmetry of objects. It decomposes the pose space into symmetry-invariant and symmetry-related parts. The method discretizes the object’s surface into fragments and predicts a probability distribution for each fragment, classifies pixels based on the associated object segments, and regresses coordinates. This approach is adaptable to various types of symmetrical objects, including those with reflective surface symmetry or rotational symmetry.

Pix2Pose [63] uses an untextured 3D model to regress pixel-level 3D coordinates from RGB images. It introduces a transformer loss function specifically designed for symmetric objects and trains a self-coding network with a Generative Adversarial Network (GAN) [64] to denoise the model and recover occluded parts. The method has been evaluated on the T-LESS dataset [12]. It uses the visible surface deviation as a metric, which measures only the distance error of the visible parts; this metric is independent of symmetry and occlusion, and the results of Pix2Pose outperform previous methods significantly. This method has good applicability in industrial-related scenarios.

RNNPose [65] proposes a pose refinement method based on Recurrent Neural Network (RNN) [66] design, using CAD models for rendering. It optimizes the error between the rendered image and the observed image using nonlinear least squares. RNNPose introduces a hybrid network trained with contrast to handle occlusion, making it more robust against errors and occlusion introduced by the initial poses. The method shows substantial improvements over the initial poses obtained from PoseCNN [20]. However, one limitation is that the training model is object-specific, and still needs to be improved if it is to meet the generalization requirements for unseen objects.

Onepose [8] presents a novel approach for 2D–3D feature matching using a graph attention network [67]. This method effectively preserves the graph structure information of feature tracking, resulting in more reliable and faster matching. It achieves a higher accuracy compared to PVNet [46], without requiring instance-specific training on the validation set. Hybridpose [68] proposes a network architecture based on PVNet [46], leveraging a prediction network with three intermediate representations using ResNet [69]. By fusing the features of keypoints, edges, and symmetry points, this approach expresses geometric knowledge with multiple intermediate representations, providing additional constraints on the object. The introduction of edge and symmetry point features improves the stability of position estimation. However, it is worth noting that training the network requires careful design.

CRT-6D [70] employs a sparse set of features based on key points on the object’s surface, significantly reducing the impact of noise and computational cost. This method incorporates a fast refinement technique for better real-time performance, utilizing a deformable attention mechanism to handle occlusion robustly. However, it should be acknowledged that the accuracy of CRT-6D is lower compared with the approach proposed in Zebrapose [36].

Among the feature-based methods, pose estimation using the PnP algorithm is widely adopted, and exploring ways to improve the PnP algorithm is also a direction. Previous approaches [25] have employed various techniques such as direct usage of the PnP algorithm, the EPnP [71] method [46], or combining PnP with RANSAC [45].

However, the non-differentiability of the PnP problem at some points poses challenges for convergence during training. To address this issue, the CVPR2022 Student Best Paper, EPro-PnP [72], tackles the problem of solving the camera pose by transforming it into a probability density prediction task. By learning the 2D–3D correlation based on the ground truth pose, EPro-PnP achieves end-to-end training of a network that predicts the probability density of the pose. This approach not only solves the PnP pose optimization problem, but also provides insights for optimizing other networks. It enables stable and flexible training of pose estimation networks based on PnP geometry optimization, surpassing the state-of-the-art performance of the outdated CDPN [73] method.

EPro-PnP [72] essentially applies the multi-class softmax concept to the continuous domain, which can be extended not only to other geometric optimization-based 3D vision problems [40], but also theoretically generalized to train general models with nested optimization layers.

In general, the method based on global feature matching offers advantages in terms of speed, while the method based on local feature matching better fulfills the accuracy requirements. Global features rely heavily on semantic information, providing stronger discriminative capabilities, while local features rely more on texture information, making them more robust to image variations. The sparse counterpart of feature-based methods requires less computational resources and can perform better in some real-time applications. It offers an overall satisfactory performance. In contrast, the dense counterpart utilizes richer information and is particularly effective at handling occlusion problems. However, it demands higher computational resources. Feature-based methods, in general, are fast and robust, especially when dealing with texture-rich objects. However, they may be less effective when applied to weakly textured objects, where the distinction between objects and the background is weak, and detecting keypoints becomes challenging.

#### 2.1.4. Refinement Methods

**Figure 7:**
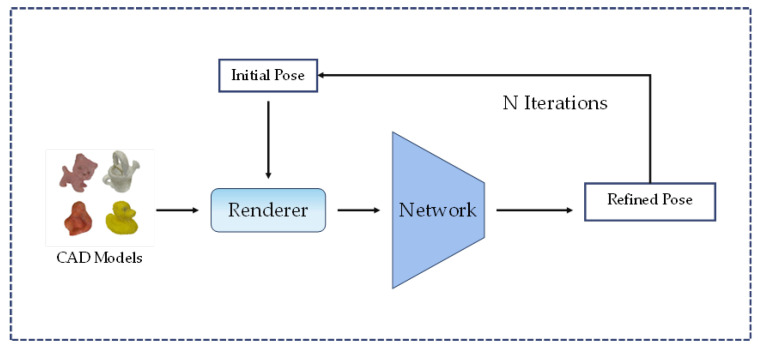
General flow of the refinement method.

Refinement methods play a crucial role in improving the performance of pose estimation by refining the initial coarse pose. RGB-based methods often require subsequent optimization, and several popular approaches have been proposed [33,34,65,74]. Figure 7 shows a simplified flow of the refinement method using the renderer.

PoseCNN [20,75] utilizes the Iterative Closest Point (ICP) algorithm to align known models with the depth map for pose refinement. DeepIM [33] takes an iterative approach, using a pose refinement network to minimize the difference between the observed image in the current pose and the rendered image. Another method [76] introduces a novel visual loss for pose updating, which aligns contours to refine the pose. HybridPose [68] proposes a pose refinement method that utilizes a robust norm optimization of the reprojection error, termed GM robust norm optimization.

DPOD [45] presents a pose refinement network that includes modules for independent regression of rotations and translations. It optimizes the estimation results based on the difference between the image rendered by the predicted pose and the real input. CosyPose [34] draws inspiration from bundle adjustment and globally refines all objects and camera poses by minimizing multi-viewpoint reprojection errors. Repose [74] introduces a faster refinement approach by extracting the image features using U-Net [77]. RNNPose [65] formulates pose optimization as a nonlinear least squares problem.

Some of the RGB-based methods are uniformly compared in Table 1, employing metrics including Average Distance of Detected points (ADD) and Symmetric ADD (ADD-S) on both the LineMod (LM) [78] dataset and LineMod-Occlusion (LM-O) [79] dataset, as well as the area under the curve (AUC) of ADD-S on the YCB-Video (YCB-V) dataset [20].

### 2.2. Point Cloud or Depth-Based Methods

In some situations of object pose estimation, such as in industrial environments, the limitations of RGB-based methods are evident due to the lack of color and texture information [82]. In contrast, methods based on point clouds or depth maps may offer unexpected advantages, while RGB images lack geometric data. Depth information or point cloud information contains rich shape geometry information, which is significant for inferring the pose of objects [83,84].

**Figure 8:**
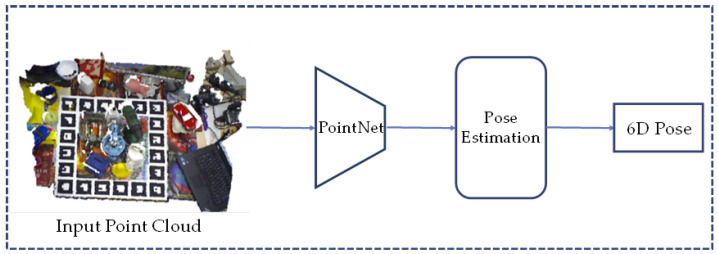
Point cloud-based typical approach flow.

Methods based on depth maps or point clouds may have advantages in training data. Methods based on real images usually require expensive manual labeling. The annotation cost can be reduced when using synthetic images, but the domain gap becomes an important issue. Methods based on depth information or point clouds have smaller domain gaps with more robust results [85]. Figure 8 shows a typical approach using point clouds as inputs.

Research on point cloud or depth maps aims to achieve a balance between accuracy and computational speed by combining global and local features, and is therefore particularly suitable for objects with different surface textures [86]. Colored point-pair features have been introduced in traditional methods [87] to improve discrimination and accuracy by exploiting the color information. There are also some works where the point cloud is used directly as an input to achieve the desired results through deep learning.

#### 2.2.1. Point Cloud-Based Methods

Liu et al. [88] proposed a new downsampling method that combines edge and geometric information to estimate complex shapes, oriented to the requirements of medicine, and a pose estimation method based on edge-enhanced point-pair features for the characteristics of the spine structure. This method showed competitiveness when dealing with complex shapes and symmetric objects and is applicable in automated surgery. However, this method may not perform as expected when dealing with tiny objects or asymmetric cylindrical objects.

Previously, 3D data faced inherent challenges when represented using 3D voxel meshes or multi-view projections, including high computational requirements and loss of geometric information. To address this problem, Pointnet [89] proposed a solution based on point cloud data. At the same time, to address the problem of disorganization of the point cloud, the method employs a simple symmetric function to aggregate the vertex information, starting with global feature extraction, and then performs point cloud segmentation or classification.

Based on Pointnet [89], the Pointnet++ algorithm [90] further improves the acquisition and processing of localized information in point clouds. Both networks play an important role in various point cloud-based tasks. Another innovative approach is Pointvotenet [91], which employs a 3D segmentation method, based on Pointnet [89], to estimate the pose directly from a disordered 3D point cloud, unlike traditional projection-based methods. However, Pointvotenet minimizes the keypoints of symmetry in the process, and there is still space to improve the performance, while in real-time demanding scenarios this may not be applicable. The RandLA-Net algorithm [92] introduces stochastic downsampling to point cloud processing, simplifying network complexity while preserving local features through a feature aggregation module.

The PointPoseNet [93] method performs segmentation and vector prediction of point clouds obtained from RGB-D images, which in turn results in optimal pose estimation. It works well for scenes in the presence of occlusions, but the runtime increases when faced with the need to process multiple instances.

Point pair features (PPF) [86] is a method of global modeling and local matching, and PPF-based methods are known for their potential to achieve a high accuracy, while they often come with the drawback of a high computational complexity. In response to this challenge, PPFNet [94] combines PPF with deep learning techniques to enhance 3D point matching and point cloud feature extraction. The experimental results demonstrate that the learned features outperform traditional methods significantly in tasks like 3D shape retrieval and matching. The deep-learning-based 3D target recognition methods show a superior generalization performance compared with traditional methods.

In real-world applications, such as the task of robot bin-picking applications, where objects are randomly stacked and occlusions in the scene are common, the Point-Wise Pose Regression Network (PPR-Net) [82] is a straightforward and effective solution. This network utilizes input point cloud data to simultaneously process instance segmentation and pose estimation, which can effectively identify occlusion relations and handle symmetric objects, thus achieving favorable results in practical applications.

Hoang et al. [95] introduced a detection method in pose estimation that relies on a voting mechanism designed for point cloud inputs without segmentation. Their method incorporates an attention module for learning rich associations between object parts and instances to improve the pose estimation performance. This method works well when dealing with datasets containing industrial parts, and it may be suitable for industrial scenarios.

CloudAAE [85] proposed a new method for reconstructing point clouds by regressing 6D poses using desired viewpoints and synthetic data based on 3D models, with temporary point locations. By augmenting the autoencoder to generate a noiseless, occlusion-free point cloud, the online approach offers advantages in terms of time efficiency and hardware storage over rendering methods. However, the method relies on the iterative closest point (ICP) algorithm [21] for optimization, which may not be optimized in the case of severe occlusions.

Point cloud-based pose estimation has important applications in robotic 6DoF grasping, and with the co-development of complete and local point cloud methods, the robustness of industrial grasping, as well as its adaptability to the environment, has improved greatly.

#### 2.2.2. Depth-Based Methods

**Figure 9:**
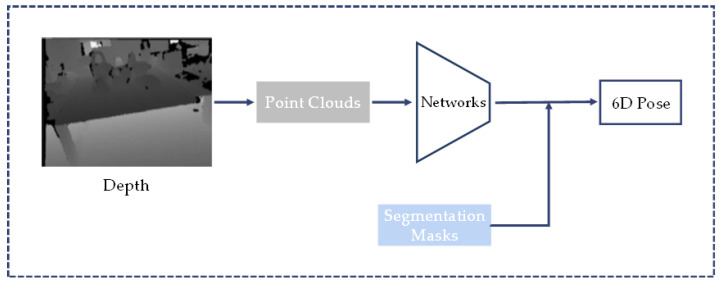
Depth-based typical approach flow.

A common method for 6D pose estimation from depth images is to convert the depth image into a point cloud, and then perform pose estimation through the obtained segmentation mask, as shown in Figure 9.

A brand new framework, SwinDePose, was proposed in [96], which extends the Swin Transformer [97] to pose estimation using depth information. The combination of the Swin Transformer and pose estimation achieves a high accuracy by fully leveraging point cloud information and vector data from the depth map. It also handles occlusion well, but the performance depends on the quality of the annotations.

OVE6D [98] is trained using purely synthetic data, estimated from a single depth map and segmentation mask, and decomposes the pose estimation task into viewpoint, in-plane rotation, and translation. It can be easily generalized without parameter optimization in new objects. It works well on the T-LESS dataset [12], but only applies to the model of the object and to cases where instance segmentation masks are readily available.

Methods based on point clouds or depth maps may encounter challenges when dealing with reflected light on the surface of objects. This reflection problem can hamper the accurate capture of the actual point cloud data of objects, thus affecting the quality of subsequent works and tasks. In addition, there are relatively few methods dedicated to point cloud or depth map-based position estimation. In current industrial applications, most of the point cloud methods are still adapted to cope with the challenges by improving the traditional methods. It is notable, however, that these networks also help support other methods. A typical example is PVNet [46], which utilizes the principles of PointNet [89]. This approach stands out for its efficient location estimation capabilities and has had a significant impact in the field.

Table 2 presents a systematic comparison of point cloud-based or depth-based methods. Evaluation metrics are consistently applied across all three datasets [20,78,79], utilizing the ADD(-S) metric for the assessment of the pose estimation performance.

### 2.3. RGB-D-Based Methods

**Figure 10:**
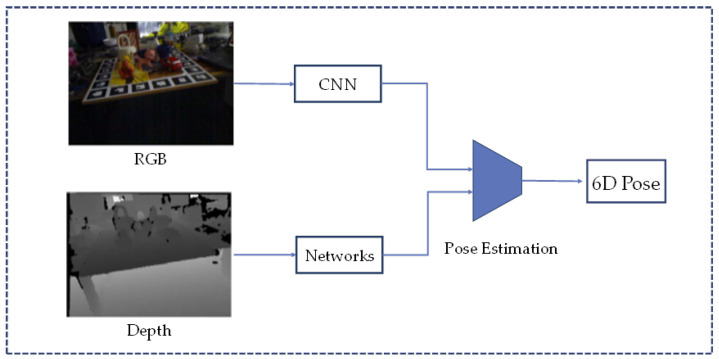
RGB-D-based typical approach flow.

Methods that rely solely on RGB images may be susceptible to challenges such as cluttered backgrounds, lighting changes, and texture differences, while methods based solely on point clouds face the problem of difficult data processing. Combining RGB images with depth information can enhance the ability to extract target geometric data, thereby improving the pose estimation performance in complex environments. Figure 10 shows a simplified flow of a typical approach using RGB-D as the input.

The main challenge in RGB-D-based methods lies in fully utilizing the appearance information from RGB images and the geometric information from depth images. Early RGB-D estimation approaches often required the extraction of information from RGB and depth images separately. For instance, in [100], pose estimation was achieved by clustering 3D feature points in the object model, allowing for the extraction of features in the object shape that are independent of perspective changes and enabling cross-view matching.

However, although cross-view methods can provide richer information, they may require a large amount of storage space [101] or require complex post-processing [20], limiting their availability for complex scenes and real-time applications. In the case of symmetric objects, it is common to restrict the range of viewpoints, necessitating additional processing steps, such as in BB8 [60] for view classification and PoseCNN [20] for average distance computation between transformed models and estimated poses. Nonetheless, the process of finding the nearest 3D point can be time consuming.

On a different note, Li et al. [102] introduced a method to incorporate depth information as an additional channel. The network was designed by combining RGB and depth data in feature channel dimensions. This method has proven to be effective for multi-object instances and handling occlusions, but it may not perform optimally in single-view scenarios. The complexity of the network structure can also result in higher time costs. In this section, RGBD-based methods are classified into three categories: fusion-based methods, keypoints-based methods, and other methods.

#### 2.3.1. Fusion-Based Methods

To tackle challenges related to occlusion and poor lighting conditions, DenseFusion [103] employs separate feature extraction and dense fusion of color and depth information. A pose estimate is generated for each pixel and the final result is obtained by voting. This approach considers the structural information of the depth channel, leading to accurate object pose estimation. Remarkably, it is nearly 200 times faster than the PoseCNN [20] with the ICP combination method. However, DenseFusion is limited to estimating the 6D pose of known objects and demands high-quality depth data and substantial computational resources.

MoreFusion [5] is tailored to scenarios where objects are known in robotics applications, focusing on solving pose estimation problems in the contact and occlusion of different objects. It achieves this by fusing segmentation masks into volumetric maps to represent occupied and free space. This approach enables pose estimation with awareness of the peripheral information, initial rough voxel reconstruction, and multi-object pose estimation, even in cases of occluded contact. Differentiable collision refinement and CAD model alignment support robot planning for grasping tasks in complex scenarios. When compared with DenseFusion [103], MoreFusion [5] also performs well in severe occlusion situation, and integrates more physics knowledge into the optimization framework.

FFB6D [104] enhances DenseFusion [103] with an improved fusion module. Fusion is applied at each coding and decoding layer to maximize the utilization of local and global information from another network. This simplifies keypoint localization and yields accurate pose estimation, resulting in a high accuracy. However, it is important to note that the effectiveness of deep fusion is highly dependent on data quality, with data noise significantly impacting performance. Furthermore, post-processing operations account for over half of the time cost.

From the above, it can be seen that the fusion-based method can utilize the two types of data more elegantly and is more robust to occlusion environments.

#### 2.3.2. Keypoints-Based Methods

**Figure 11:**
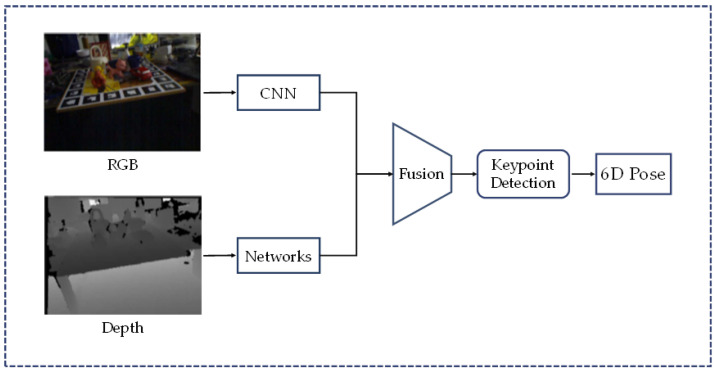
Keypoint-based typical approach flow.

Keypoints-based methods are also an influential class of methods, which achieve position prediction by finding keypoints in an object through correspondences. A typical approach to the brief flow is shown in Figure 11.

PointNet [89] serves as a solid foundation for methods like PointFusion [11]. However, it has certain limitations in effectively extracting local point cloud features. To address this, PVN3D [105] introduces a two-stage approach, encompassing a feature extraction module, a keypoint detection module, a semantic segmentation module, and a centroid voting module. These modules work together to identify key points of objects through voting and clustering. Subsequently, after detecting the 3D key points of the target, the least squares method is used to fit the pose. Notably, the combination of 3D keypoints and semantic segmentation enhances the overall performance, making the approach more robust, especially in the presence of natural occlusion.

Lin et al. [106] propose an end-to-end regression-based pose estimation method rooted in geometric information. This method supervises the decomposition of keypoint offsets into unit vectors and lengths and introduces an improved keypoint sampling strategy to ensure an adequate number of sampling points for small objects. However, it encounters challenges when addressing symmetric cases due to the lack of clear keypoint definitions for symmetric objects.

Zhou et al. [107] employ Deep Fusion Transformer (DFTr) blocks to elevate pose estimation by aggregating globally enhanced features across different modalities, facilitated by semantic similarity. They introduce a globally optimized voting algorithm to obtain accurate keypoints and exhibit robustness in dealing with various occlusions and symmetries while maintaining real-time performance. But improper selection of DFTr blocks can lead to overfitting, and the computational demands are relatively high.

The keypoints-based approach is more robust to noise and has a relatively good estimation, which makes it more practical, but it requires the determination of suitable keypoints.

#### 2.3.3. Other Methods

In addition to the two methods mentioned above, there are many other studies based on RGB-D. Addressing the issue of previous methods employing separate networks for RGB and depth information extraction, Uni6D [108] introduces a unified CNN framework based on Mask R-CNN [23]. This framework incorporates additional UV data as an input to resolve the projection decomposition problem. Uni6D stands out for its efficiency in terms of time and cost, and achieves approximate accuracy on the YCB-V [20] dataset. It is exciting that it is 7.2 times faster than the FFB6D [104] method. Nevertheless, it has to be recognized that the simplification process may lead to accuracy degradation and further research is necessary, especially when denoising RoI features.

G2L-Net [109] takes a global-to-local approach, focusing on extracting point clouds from RGB-D data through 2D detection. The network performs 3D segmentation and translation prediction based on the coarse point cloud. It also captures viewpoint perception information using point-based features. G2L-Net estimates the initial rotation in the coordinate system transformed from the fine point cloud and further enhances the accuracy by considering rotation residuals between the predicted and true values. Impressively, G2L-Net achieves a good real-time performance despite the multi-step process.

More comprehensive utilization of geometric information has been shown to help mitigate issues related to color and appearance interference, random occlusions, and generalization from unseen instances. Previous methods that leverage geometric information often exhibit weak explanatory and generalization capabilities. In response to this, StablePose [110] introduces the concept of geometric stability to 6DoF pose estimation for the first time. Operating by the geometric stability principle, Stablepose [110] stands apart from approaches like EPOS [62], which involve sampling from a template model and regressing it as the 3D coordinates of image pixels. Instead, Stablepose learns the pose by focusing on geometrically stabilized portions of the point cloud derived from depth images, particularly emphasizing planar and cylindrical information. It accomplishes this by utilizing a minimum of three patches and predicting the pose for each patch through a sub-network. This approach significantly enhances the robustness of pose estimation in occluded scenes, as well as in objects that are not fully visible.

Building on the principles of EPOS [62], SurfEmb [16] presents a technique to learn a continuous dense distribution with the aid of contrast loss. This allows the model to capture multimodal distributions on an object’s surface, making it more effective at handling symmetry and representing positional ambiguity. However, it is important to note that the position optimization process may encounter challenges when surface changes are subtle, and the approach involves four stages.

MegaPose [111] proposes a method that provides pose estimation of novel objects from RGB or RGB-D images. Rough pose estimation is performed first by classification and then refined by rendering synthetic views, which is simple to couple with other detection methods. The method is tested for its performance on multiple datasets and is suitable for real robots operating on unknown objects, but the runtime needs to be considered and not all rough initial poses can be successfully refined.

Lipson et al. [112] propose an end-to-end network that utilizes geometric knowledge to refine the pose and correspondence through coupled iterations and dynamically reject outliers. This method uses a novel bidirectional PnP algorithm, where the entire network can learn to optimize and perform pose updates. The refinement method may result in a less effective local optimal solution when the initial pose rotation error turns out to be large. This method also works well when only RGB images are used as the input.

Numerous pose estimation methods, including those referenced in [16,33,36,103], require object detection methods. However, in complex scenes with a poor detection performance, the estimation results of these methods will be greatly affected. To address this issue, Hai et al. [113] introduce a rigidity-aware detection method. This innovative approach capitalizes on the inherent rigidity property of the task object and formulates bounding boxes by sampling from the visible region rather than including the occluded part. The robustness of the target detection is enhanced, and the detection results can further improve the pose estimation effect.

The methods with RGB-D input offer several advantages and disadvantages. On the positive side, the combination of color information and depth information allows for more accurate and robust pose estimation, particularly in challenging scenarios with occlusion or poor lighting conditions. The depth data provide valuable geometric information, enhancing the recognition and localization of objects. Additionally, this approach can be instrumental in real-world applications such as robotics, where precise pose estimation is crucial. However, there are some drawbacks to consider, including increased computational demands due to processing both RGB and depth data.

In summary, RGBD-based methods provide a unique advantage by harnessing the synergy of RGB and depth information, resulting in a substantial improvement in estimation accuracy. How to effectively utilize different data is the key to these methods.

Table 3 presents a comparison of some RGBD-based methods. Evaluation metrics are consistently applied across all three datasets: LM, LM-O, and YCB-V [20,78,79], utilizing the ADD(-S) metric for the assessment of pose estimation performance.

## 3. Category-Level 6DoF Object Pose Estimation

Category-level 6D object pose estimation is designed to predict the complete pose of rotations, translations, and dimensions of object instances observed in a single arbitrary view of a cluttered scene. Estimating the pose and shape of daily objects is also an essential task, the majority of the previously discussed instance-level methods rely on accurate CAD models, but in daily life environments, it is hard to obtain CAD models of objects in advance, whereas category-level pose estimation methods aim to estimate the poses of arbitrary shapes in the same category without a priori assumptions of known CAD models, and it is starting to attract more attention by dealing with multiple instances of real-life scenarios [116,117,118].

### 3.1. Regression-Based Methods

**Figure 12:**
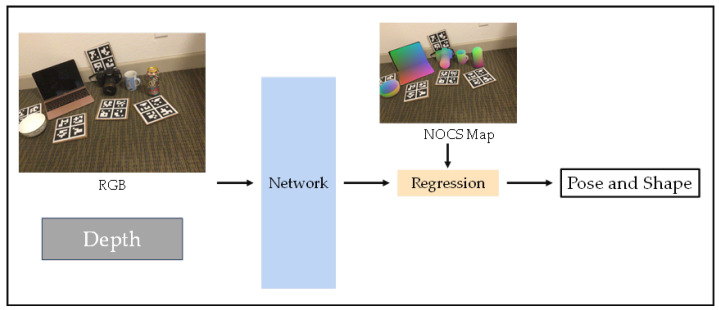
Typical flow of regression-based category-level pose estimation methods.

The regression-based approach is a single-stage approach—one of the most straightforward. A simplified flow diagram of a typical process for such methods is illustrated in Figure 12.

Category-level pose estimation faces challenges due to the unavailability of ground truth data. NOCS [116] addresses this by introducing a context-aware mixed reality approach, and it can be considered a pioneering work. To handle different and unseen object instances within a category, NOCS proposes a Normalized Object Coordinate Space (NOCS) and a dataset frequently used in category-level pose estimation tasks. Additionally, to cope with the symmetry of real-life objects, an axis of symmetry is defined for each category in the training data, ensuring that predefined rotations result in consistent loss values. While this approach enables robust pose and size estimation for unseen objects in real environments through direct regression, forming a uniform within-category representation remains challenging.

DualPoseNet [119] introduces a novel approach by stacking both an implicit and an explicit pose decoder on a shared pose encoder. This architecture allows for complementary supervision during training, ensuring the consistency of the predicted pose between the two decoders through an adaptive loss term. To further enhance its capabilities, DualPoseNet incorporates a spherical fusion module designed to facilitate more efficient learning from the input appearance and shape features. This method predicts a more compact bounding box, achieves more accurate pose estimation results, and performs well on instance-level tasks.

For addressing intra-class object variations, FS-Net [17] introduces a novel data augmentation method designed to enhance efficient feature extraction. In the context of category-level pose estimation, this approach proves valuable for handling objects with diverse shapes. This method uses only a limited amount of real data for training, demonstrating its proficiency in efficiently extracting category-level features from a small dataset. It proves effective for tasks characterized by a limited number of samples, but its success relies on the use of a high-performance and robust detector.

Centersnap [120] adopts a unique perspective by considering objects as spatial centers, where each center encapsulates a complete representation of an object’s shape and pose. Notably, objects within the same category consistently retain the same semantics, even when their shapes are different. This approach is less demanding on computational resources and is better suited for tasks with real-time requirements.

Networks of direct regression methods are easier to deploy, although somewhat less able to cope with change. The subsequent emergence of methods that utilize prior knowledge can significantly improve the overall performance.

### 3.2. Prior-Based Methods

**Figure 13:**
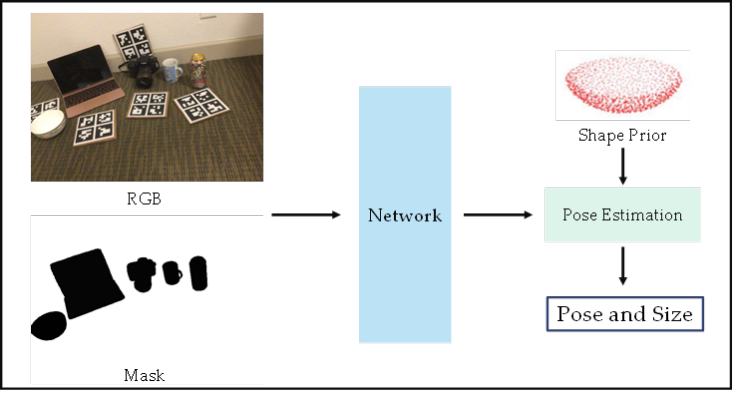
Typical flow of prior-based category-level pose estimation methods.

Exploiting the prior knowledge in category-level pose estimation tasks can be an effective method. By incorporating the prior knowledge learned in the provided instances, more accurate estimates can be obtained. SPD [121] has introduced a prior-based framework to tackle intra-class variation, which has become one of the mainstream approaches. The simplified flow of the method based on prior knowledge is shown in Figure 13.

Subsequent work, ACR-Pose [122], emphasizes the importance of reconstructing canonical NOCS representations. ACR-Pose employs an adversarial training scheme consisting of a reconstructor and a discriminator to improve the network’s ability to reconstruct high-quality canonical representations, enhancing the estimation accuracy, especially in challenging intra-class scenarios. The use of adversarial reconstruction loss has influenced subsequent category-level pose estimation methods, seeking to overcome inherent intra-class variation. It is perhaps less applicable for scenarios where occlusion is common, as this method may fail in cases of occlusion or truncation.

SGPA [123] introduces a canonical prior model with shape deformation for pose estimation, further enhancing the correlation using structural similarity dynamics adaptation. However, the prediction based on the prior model may be less accurate when dealing with significant shape differences between categories.

To minimize the effect of domain gaps caused by the use of synthetic data, DPDN [124] proposes a method based on a deep prior deformation network, which introduces a self-supervised objective through a type of coherent learning to improve the sensitivity to pose changes. The method is suitable for situations where synthetic images are used for training.

RGB features are sensitive to color variations. In contrast, the introduction of additional shape prior features makes the results more robust, and methods based on prior knowledge are becoming popular.

### 3.3. Other Methods

In addition to the above main methods, there have been many other methods [8,125,126,127] for category-level pose estimation in recent years. To address the potentially high computational costs of complex multi-stage methods, Li et al. [125] leverage RGB-D images for single-stage object pose and shape estimation. Their method utilizes semantic primitives within a generative model to allow semantic features to model diverse shapes and establish connections between the observed point cloud and implicitly generated shapes. The optimization of an object’s shape in arbitrary poses is achieved by using a novel SIM(3) invariant descriptor, delivering superior optimization results. However, it is important to note that this method may not account for occlusion and could result in ambiguous single-view estimations. Furthermore, utilizing implicit representations for shape inference is more complex than direct regression of shape parameters.

Chen et al. [126] introduce a method that leverages category information without relying on CAD models. Their approach involves synthesizing object images from various viewpoints using a generative adversarial network, combining a gradient-based fitting process with a parametric neural image synthesis module. This module can implicitly represent the appearance, shape, and pose of an entire object class, which eliminates the need for explicit CAD models for individual object instances. Notably, using only RGB images as inputs, this method can accurately recover the orientation information. However, achieving full 6DoF poses necessitates the incorporation of additional depth information to overcome scale ambiguity.

OnePose [8] adopts the concept of visual localization, exclusively using RGB images without reliance on CAD models. It can construct representations of specific objects from simple video scans using only a few samples. This unique approach allows it to handle objects from any class without the necessity for instance- or class-specific network training. OnePose excels in delivering fast and accurate position estimation, all without the need for prior knowledge. However, it is worth noting that the method relies on local feature matching and may encounter challenges when dealing with untextured objects.

Lin et al. [127] propose a keypoint-based single-stage method for category-level pose estimation only using a single RGB image as the input. Few studies have been conducted for this task only through RGB images before this approach. This method detects the target object from the input image and then performs pose estimation by predicting the 3D bounding box projection in conjunction with PnP, and finds that accurate bounding box size prediction is critical for category-level tasks. It has notable potential for robotics applications.

Table 4 and Table 5 show performance comparison results of the category-level position estimation methods on the NOCS dataset [116].

## 4. Datasets and Metrics

### 4.1. Datasets

**Figure 14:**
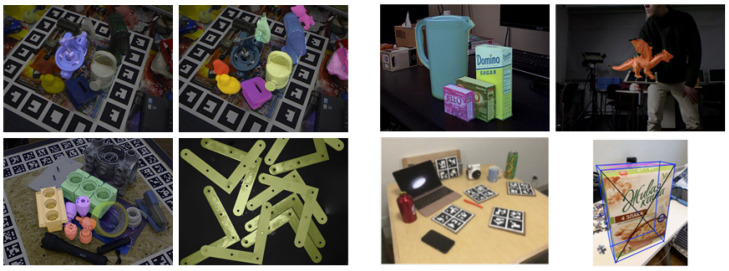
Examples of some datasets. (**Left**) Examples of LM, LM-O, T-LESS, and ITODD datasets from BOP. (**Right**) Examples of YCB-V, TUD-L, NOCS and Objectron datasets from BOP.

Deep-learning-based methods greatly benefit from access to extensive and high-quality training data. In this section, we provide an overview of some of the most commonly used and representative datasets in object position estimation tasks, categorize them according to whether they belong to the instance level or the category level, and describe the application scenarios for which each dataset is suitable. The BOP Challenge [35] serves as a pivotal initiative that organizes multiple 6DoF pose estimation datasets into a standardized format. It is also classified according to whether the dataset belongs to the instance level or the category level, and the application scenarios suitable for each dataset are described. This unification not only simplifies the evaluation of various pose estimation methods, but also fosters significant advancements in the development of deep learning-based pose estimation techniques. The part of the used datasets shown in this paper comes from the BOP website at https://bop.felk.cvut.cz/datasets/ (accessed on 1 February 2024). For a detailed comparison of these datasets and their applicable scenarios, please refer to Table 6. The sample presentations of some of the datasets are shown in Figure 14.

LineMOD (LM) [78], introduced by Hinterstoisser et al. at the 2012 ACCV conference, stands as one of the most widely utilized datasets in the field of 6DoF pose estimation tasks. It also plays a role in object detection tasks. The dataset contains 15 categories of daily objects and comprises more than 18,000 real images, each accompanied by finely labeled poses. LineMOD is suitable for pose estimation in cluttered scenes with minor occlusions.

Occlusion LineMOD (LM-O) [79], introduced by Brachmann et al. at ECCV 2014, has been proposed to meet the requirements of severely occluded scenes. This dataset extends a test set from LineMOD (LM) and involves photographing objects under three different lighting conditions, introducing significant occlusion across eight object categories.

T-LESS [12], introduced by Hodan et al. at the WACV conference in 2017, is designed for the challenging task of 6DoF pose estimation, particularly focusing on textureless objects. This dataset contains 30 industrially relevant objects that lack apparent texture and color information. These objects share similarities in shape and size, and exhibit symmetry, which pose significant occlusion challenges when multiple objects are combined. T-LESS includes 20 scenes of varying complexity and provides texture-free CAD models for each object. Overall, T-LESS is a very challenging dataset in the 6DoF pose estimation task.

YCB-V [20], introduced in the context of PoseCNN, represents an extension of the YCB dataset. This dataset comprises 21 objects characterized by adjusting the shapes and textures, and it is derived from 92 videos that encompass scenarios with occlusion and different symmetries. These variations are influenced by image noise and diverse lighting conditions. YCB-V includes a combination of both real and synthesized images, making it suitable for application scenarios that involve daily scenes and 6DoF pose estimation based on video sequences.

ITODD [13], introduced by Drost et al. at ICCVW 2017, is a dataset that contains 28 objects photographed in real industrial environments. This dataset specifically focuses on mechanical parts within industrial settings, where color information is often limited. Notably, each scene in ITODD is captured using two industrial sensors and three grayscale cameras, which results in a high-quality dataset that offers valuable 3D industrial scenes.

TUD-L and TYO-L [35], both introduced in the 2018 BOP Challenge [35], offer distinctive pose estimation datasets designed for various environments and illumination conditions. TUD-L comprises datasets featuring three moving objects, subjecting these objects to eight distinct illumination conditions in the test images. Meanwhile, TYO-L includes 21 objects captured on four different tablecloths and under five diverse illumination conditions. A notable feature of these datasets is their applicability to scenes with varying lighting conditions encountered in daily life.

NOCS [116], which stands for Normalized Object Coordinate Space, was proposed by He Wang et al. in 2019 for Category-Level 6D Object Pose and Size Estimation. It comprises six object categories, including bottle, bowl, camera, can, laptop, and mug, along with a distractor category. The NOCS dataset contains 31 indoor scenes and is divided into two sub-datasets: the real dataset REAL25 and the virtual dataset CAMERA275. Notably, a significant portion of current category-level pose estimation research relies on this dataset.

Siléane dataset [14], introduced in 2017, provides RGB-D images alongside corresponding semantic segmentation labels. It serves as a small yet finely labeled semantic segmentation dataset focused on outdoor scenes, and is provided with different symmetries in eight object categories.

The Fraunhofer IPA Bin-Picking dataset [137], introduced in 2019, contains 10 categories of objects and includes depth maps, point clouds, and RGB maps. This dataset offers large-scale data designed for complex industrial scenarios and multiple parts for industrial grasping. It extends the scope of the Siléane dataset [136] to cover more diverse scenarios suitable for deep learning. Additionally, it introduces two new industrial object categories.

Shapenet [135], proposed in 2016, is a point cloud dataset that comprises 16 large categories and 55 small categories commonly found in daily life. Each large category includes lots of model data, with multiple models corresponding to each category. Shapenet provides various semantic annotations for each model, which supports the segmentation of different instances of parts and is widely utilized in a range of visual tasks based on point clouds.

Objectron [138], proposed in CVPR 2021 by Ahmadyan et al. Contains nine categories of objects with 4 million labeled images from 14,819 videos. The dataset is designed for category-level pose estimation, with each category consisting of hundreds of examples captured under different lighting conditions. Significantly, these videos showcase stationary objects from various perspectives, consistently providing bounding boxes, also making them well-suited for tracking tasks. The data collection took place in the wild environment, enhancing its real-world generalizability.

### 4.2. Metrics

Different algorithms can be evaluated more fairly under the same evaluation metrics, and the following evaluation metrics are commonly used in 6DoF pose estimation:

ADD (Average Distance of Model Points): ADD measures whether the average deviation of the transformed model points is less than a certain value of the diameter of the object. The commonly used index value is ADD-0.1d, and it is considered that the estimation is correct when the distance is less than 10% of the size of the model diameter.

2D Projection Metric: This metric calculates the average distance between the projections of the 3D model points given the estimated pose and the ground truth pose. If the distance between projections is less than five pixels, the pose is considered correct. Note that a CAD model of the target object needs to be known for this metric.

n◦n cm Metric: The effectiveness of the pose estimation is tested by measuring the rotation angle and translation distance errors. A common metric is 5°5 cm, meaning that the pose estimation is considered correct if the absolute value of the error of each rotation angle does not exceed 5°, and the absolute value of the error of the translation position from the real data does not exceed 5 cm. Additionally, 5°10 cm and 10°10 cm are commonly used as numerical settings. For symmetric objects, the ADD (-S) metric is used, i.e., the distance to the closest point is used instead of the average distance calculation.

VSD (Visible Surface Difference): VSD considers only the visible object part and treats indistinguishable poses as equivalent. It is applicable for symmetric and occlusion cases. The higher the overlap between the estimated pose and the true value in the visible region, the lower the error. This metric is often used in research work on the T-LESS dataset.

MSSD (Maximum Symmetric Surface Distance): MSSD measures the maximum distance deviation of a surface point measured in 3D space. It is relevant for robotics applications. The smaller the maximum distance deviation between the surface of the 3D object in the estimated pose and the surface points in the true value pose, the smaller the error.

MSPD (Maximum Symmetric Projection Distance): MSPD measures the maximum deviation perceivable on the image plane and is relevant for augmented reality applications. It calculates the maximum deviation of the 2D profile of an object on the image plane. It is similar to MSSD, but calculates 2D projection. The smaller the maximum deviation of the 2D contour in the estimated pose from the true pose contour, the smaller the error.

AR (Average Recall): In the BOP Challenge [35], the pose error is measured by the average of three error functions: Visible Surface Difference (VSD), Maximum Symmetric Surface Distance (MSSD), and Maximum Symmetric Projection Distance (MSPD).

## 5. Analysis and Possible Future Directions

### 5.1. Analysis of Task

Convolutional neural networks, are good at establishing mapping relationships between 2D images to 3D objects, and methods utilizing deep learning of the networks have been shown to significantly improve pose estimation accuracy and robustness [139,140,141]. It can be known from the results of Table 1 and Table 3 that, in general, the same method framework performs better in terms of accuracy when based on RGB-D, but it has an efficiency advantage when based on RGB images only. As shown in Table 7, based on the previous content, we summarize the applicable scenarios and limitations of different algorithms.

Estimating rotations is significantly more complex and difficult than estimating translations. Common rotation representations, such as rotation matrix, quaternion, and Euler angle, are usually discontinuous in 3D Euclidean space, which is very challenging for neural network training. Sundermeyer et al. [75] utilize autoencoders to learn implicit 3D orientation features directly from images, embedding rotation information in the latent representation. Zhou et al. [142] introduce a continuous representation definition that is of great benefit to deep-learning-based methods.

According to the characteristics of different data sets in Table 6, in industrial applications, metal parts often lack color information, the surface shows different reflections under different lighting conditions, and the objects are mostly symmetric, with smooth surfaces and less texture information. In these situations, methods based on RGB images may not be so effective, the use of RGB-D or point cloud data needs to be considered. Symmetry is also a widespread problem in pose estimation tasks. To handle it, methods such as defining a suitable loss function [20,63], utilizing geometric constraints and transformations designed for rotationally symmetric objects [60], or employing multi-view fusion [34] can be considered.

While in daily life scenarios, CAD models or depth maps are not readily available for many objects, color images are very easy to capture. Here, it is possible to consider using only RGB images as the input or a category-level method. Compared with instance-based methods, category-level pose estimation methods have a better generalization ability to different shapes when category information is known. From the information in Table 4 and Table 5, we can know that there is still much room for improvement in category-level pose estimation.

When considering training samples, tools for labeling object poses are provided by Label Fusion [143], among others. However, labeling 6D poses in real images is both expensive and unavoidably subject to a not insignificant percentage of errors [16,61]. In contrast, synthetic images are advantageous due to their low time cost and storage efficiency. Some approaches [32,38] utilize synthetic images for training or explore self-supervised learning methods to deal with the problem that labeling real images is difficult. However, the drawbacks of synthetic-to-real must not be overlooked, and the domain gap generated by only training on synthetic images could affect their use in real-world scenarios. To address this, referencing techniques used in the BOP Challenge [144], which generate synthetic images through physically based rendering methods, can help minimize the domain gap between synthetic and real images. Also, the treatment of DPDN [124] in category-level pose estimation can be referenced.

The refinement method has proven very effective for initially rough poses. There are already some methods that improve accuracy through refinement, the usage of PoseCNN [20] results in DeepIM [33] and the combination of Repose [74] and PVNet [46] are successful examples. However, while refinement enhances accuracy, it also entails some efficiency trade-offs for the method.

To meet the real-time requirements of the task, an end-to-end pose estimation method based on sparse feature matching can be considered, or a method combined with detection results can be designed. Generally, sparse feature matching methods are faster than dense matching. Compared with segmentation-based methods, object detection-based methods can better meet the speed requirements of processing before pose estimation. But time efficiency is not the only goal, multi-stage approaches may be more time-consuming than end-to-end approaches, but each module can be optimized independently to improve accuracy and are easier to modularize for different specific tasks.

### 5.2. Challenges and Possible Future Directions

In recent years, driven by the rapid development of computer vision, deep-learning-based pose estimation methods have made significant progress. However, these methods still face various challenges and a lot of research space exists.

One of the widespread challenges in various application scenarios is how to achieve accurate pose estimation under low texture, severe occlusion, cluttered background, or changing lighting conditions. To solve these problems, previous methods have made many efforts. For example, using RGB-D images may address the shortcomings of only using RGB or point clouds. Or incorporating geometric constraints and domain-specific knowledge into the network architecture and loss function design to enhance the model’s ability to utilize prior information. But there is still room for improvement.

Another challenge is the need for methods based on small samples, or even zero samples, to estimate the pose of new objects, and how to improve the generalization performance of the method. Achieving accurate pose estimation for specific object instances often demands extensive training data, limiting generalization capability. Therefore, a significant trend in this field is the development of approaches that address small sample challenges. One promising approach is category-level pose estimation, which does not rely on specific objects, can be trained with a smaller sample size, and offers robust generalization to unseen instances. Data augmentation techniques, such as symmetry transformations and illumination variations, contribute to enhanced model robustness against pose and appearance variations. Bridging the gap between synthetic and real images is an ongoing challenge in this context, and Blenderproc’s method [144] offers a valuable reference.

The third possible direction is that when facing tasks with high accuracy or speed requirements, integration with other advanced knowledge may be required. There are an increasing number of methods that use object detection [145] or segmentation methods [146] in the initial stage of pose estimation. For example, SAM [146] can be effectively utilized during the training process of POPE [147]. Convolutional Neural Networks (CNNs) excel at capturing local information, while transformer architectures [148] are adept at handling global information. Although transformers have gained prominence in tasks like human body pose estimation, their adoption in 6DoF pose estimation is relatively limited. Transformer has shown excellent performance in many areas, which means that combining CNN and Transformer may lead to better performance. While CNNs currently dominate the landscape of network architectures, recent efforts [96,107,149,150] have explored the integration of attention mechanism modules, yielding promising results. Transformers have also been used by several methods [151,152,153] to address structural irregularities present in point cloud data.

The fourth is to use multi-view information. Important factors such as physical and semantic information in the same scene are shared in different views, and better representation can be obtained by utilizing multiple views [154]. In the field of deep-learning-based pose estimation, the limitations of a single perspective are gradually emerging. The utilization of multiple viewpoints is poised to be an important direction for the future of pose estimation. These multiple viewpoints offer a more comprehensive representation of the target object, effectively alleviating visual ambiguities, and multi-view data are easily accessible in tasks such as industrial object manipulation. [155] aggregats 2D–3D Distributions of Multiple Views for Initial Position Estimation and Refinement One promising avenue involves the learning of optimal observation viewpoints. For instance, Gen6D [156] initially extracts target region features through a dedicated target detector and subsequently employs a viewpoint selection module to match these features, pinpointing the reference viewpoint most akin to the target viewpoint. This process simplifies regression challenges. Alternatively, different views can be harnessed to iteratively optimize pose estimation. By initially conducting coarse pose estimation using global features and then fine-tuning it through the alignment of detailed features from various viewpoints, a step-wise refinement of the pose is realized. This progression, from coarse to fine, has practical applications in tasks such as robot grasping, where multi-view data can inform the selection of optimal grasping positions, enhancing overall operational efficiency [157].

The final possibility is to propose new datasets to meet the demands of the task for changes in usage scenarios. Existing datasets, as shown in Table 6, while widely used, may have limitations in terms of scenario diversity and data types. To cater to the varied demands of applications across different scenarios, future datasets with richer scenarios and diverse data types are expected to emerge. Such datasets will play a key role in advancing the development of a unified framework for cross-modal pose estimation, accommodating a wide range of usage requirements.

### 5.3. Conclusions

In this study, we categorize the 6DoF pose estimation methods into two groups: instance-level and category-level. We analyze the applicable scenarios of different methods in each category, and also provide method recommendations based on the challenges faced by different application scenarios. Although the 6DoF method has developed rapidly in recent years, there is still a lot of room for in-depth research. This article also provides simple suggestions for possible future research directions. In the future, we would like to extend this work to the video field, as well as real-time pose estimation and robot grabbing.

## Figures and Tables

**Table 1:** RGB-based pose estimation methods and results.

Methods	Years	Input	Types	LM	LM-O	YCB-V
PoseCNN [20]	2017	RGB	Regression	-	24.9	61.3
SSD-6D [43]	2017	RGB	Refinement	79	-	-
YOLO-6D [25]	2018	RGB	Regression	55.95	-	-
DeepIM [33]	2018	RGB	Refinement	88.6	55.5	81.9
Deep-6DPose [22]	2018	RGB	Regression	65.2	-	-
BB8 [60]	2018	RGB	Refinement	43.6	-	-
PVNet [46]	2018	RGB	Feature	86.27	40.77	73.4
Hu et al. [24]	2019	RGB	Regression	-	27.0	-
CDPN [40]	2019	RGB	Feature	89.86	-	-
DPOD [45]	2019	RGB	Template	95.2	47.3	-
Pix2Pose [63]	2019	RGB	Feature	72.4	32.0	-
Efficientpose [41]	2020	RGB	Regression	97.35	83.98	-
CosyPose [34]	2020	RGB	Regression	-	-	84.5
LatentFusion [44]	2020	RGB	Template	87.1	-	-
Hybridpose [68]	2020	RGB	Feature	91.3	47.5	-
GDR-Net [15]	2021	RGB	Regression	93.7	62.2	84.4
SO-Pose [80]	2021	RGB	Feature	94.0	62.3	83.9
RePose [74]	2021	RGB	Refinement	96.1	51.6	82.0
PoseRBPF [47]	2021	RGB	Template	79.76	-	-
Zebrapose [36]	2022	RGB	Regression	-	76.9	85.3
RNNPose [65]	2022	RGB	Refinement	97.37	60.65	83.1
DPOD-v2 [81]	2022	RGB	Feature	93.59	-	-
EPro-PnP-v2 [72]	2023	RGB	Feature	96.36	-	-
Hai et al. [37]	2023	RGB	Regression	92.2	65.4	-
CRT-6D [70]	2023	RGB	Feature	-	66.3	87.5

**Table 2:** Point cloud or depth-based pose estimation methods and results.

Methods	Years	Input	LM	LM-O	YCB-V
Gao et al. [99]	2020	D	-	-	94.7
Pointvotenet [91]	2020	Point Cloud	96.3	75.1	-
CloudAAE [85] + ICP	2021	Point Cloud	95.5	66.1	94.0
OVE6D [98]	2022	D	96.4	70.9	-
Depth-Based [96]	2023	D	97.5	77.1	-

**Table 3:** RGB-D-based pose estimation methods and results.

Methods	Years	Input	LM	LM-O	YCB-V
Li et al. [102]	2018	RGB-D	-	-	94.3
DenseFusion [103]	2019	RGB-D	94.3	-	91.2
Morefusion [5]	2020	RGB-D	-	-	91.0
PVN3D [105]	2020	RGB-D	99.4	70.2	91.8
G2L-Net [109]	2020	RGB-D	98.7	-	92.4
PR-GCN [114]	2020	RGB-D	99.6	65.0	95.8
FFB6D [104]	2021	RGB-D	99.7	66.2	92.7
Uni6d [108]	2022	RGB-D	-	-	88.8
E2EK [106]	2022	RGB-D	99.8	75.3	94.4
RCVPose [115]	2022	RGB-D	99.4	70.2	95.2
Deepfusion [107]	2023	RGB-D	99.8	77.7	94.4

**Table 4:** Performance of category level methods on REAL275 dataset.

Methods	Years	Input	5°5 cm	10°5 cm	IoU50	IoU75
NOCS [116]	2019	RGB-D	10.0	25.2	78.0	30.1
SPD [121]	2020	RGB-D	21.4	54.1	77.3	53.2
6-PACK [128]	2020	RGB-D	33.3	-	-	-
DualPoseNet [119]	2021	RGB-D	35.9	66.8	79.8	62.2
FS-Net [17]	2021	RGB-D	28.2	60.8	92.2	63.5
ACR-Pose [122]	2021	RGB-D	36.9	65.9	82.8	66.0
SGPA [123]	2021	RGB-D	39.6	70.0	80.1	61.9
CAPTRA [129]	2021	D	62.16	-	-	-
DPDN [124]	2022	RGB-D	50.7	78.4	83.4	76.0
CATRE [130] + SPD	2022	RGB-D	54.4	73.1	-	43.6
CR-Net [131]	2021	RGB-D	34.3	47.2	79.3	55.9
RBP-Pose [132]	2022	RGB-D	48.1	79.2	-	-
SSP-Pose [133]	2022	RGB-D	44.6	77.8	82.3	66.3
GenPose [134]	2023	D	60.9	84.0	-	-

**Table 5:** Performance of category level methods on CAMERA25 dataset.

Methods	Years	Input	5°5 cm	10°5 cm	IoU50	IoU75
NOCS [116]	2019	RGB-D	40.9	64.6	83.9	69.5
SPD [121]	2020	RGB-D	59.0	81.5	93.2	83.1
DualPoseNet [119]	2021	RGB-D	70.7	84.7	92.4	86.4
ACR-Pose [122]	2021	RGB-D	74.1	87.8	93.8	89.9
SGPA [123]	2021	RGB-D	74.5	88.4	93.2	88.1
CATRE [130] + SPD	2022	RGB-D	80.3	89.3	-	76.1
CR-Net [131]	2021	RGB-D	76.4	87.7	93.8	88.0
RBP-Pose [132]	2022	RGB-D	79.6	89.5	93.1	89.0
SSP-Pose [133]	2022	RGB-D	75.5	87.4	-	86.8
GenPose [134]	2023	D	84.4	89.6	-	-

**Table 6:** Comparison of object pose estimation datasets.

Dataset	Years	Levels	Categories	Suitable Scenes
LM [78]	2012	Instance-Level	15	Objects are cluttered and untextured with limited viewpoints.
LM-O [79]	2014	Instance-Level	8	Objects are cluttered and more severely occluded.
Shapenet [135]	2016	Category-Level	16	Point cloud dataset of common objects in life with fine segmentation.
T-LESS [12]	2017	Instance-Level	30	Industry-related scenes with few object textures, strong symmetry, and mutual occlusion.
ITODD [13]	2017	Instance-Level	28	Industrial scenes with strong and scarce color information in the case of random projections.
Siléane [136]	2017	Instance-Level	8	Different symmetry objects.
YCB-V [20]	2018	Instance-Level	21	Daily objects with occlusion in different light situations, and applicable to the video needs of the object.
TUD-L/TYO-L [35]	2018	Instance-Level	24	Different light conditions.
NOCS [116]	2019	Category-Level	6	Category-level position of common objects, meet real and synthetic dataset requirements.
Fraunhofer [137]	2019	Instance-Level	10	Industrial large-scale dataset, including different modalities, is suitable for grasping tasks.
Objectron [138]	2021	Category-Level	9	Meeting generalizability and tracking task requirements with large-scale multiple views.

**Table 7:** Comparison of pose estimation algorithms based on deep learning.

Methods	Level	Advantages or Applicable Scenarios	Limitation
Regression-based methods	Instance-level	Simple design and wide application.	Applicability to complex environments may be limited.
Feature-based methods	Instance-level	Situations with rich features and not severe occlusion.	Symmetry needs to be considered.
Fusion-based methods	Instance-level	Industrial applications, are suitable for occlusion.	The method design is relatively complex.
Point cloud-based methods	Instance-level	Robot grabbing-related tasks.	Surface reflections may result in poorer results.
Regression-based methods	Category-level	Everyday objects, perform better in generalization.	Poor handling of intra-category differences.
Prior-based methods	Category-level	More robust to intra-class differences and color changes.	High demand for computing resources.

## Data Availability

No new data were created or analyzed in this study. Data sharing is not applicable to this article.
